# Legionellosis risk—an overview of *Legionella* spp. habitats in Europe

**DOI:** 10.1007/s11356-022-22950-9

**Published:** 2022-09-26

**Authors:** Piotr Kanarek, Tomasz Bogiel, Barbara Breza-Boruta

**Affiliations:** 1 Department of Microbiology and Food Technology, Faculty of Agriculture and Biotechnology, Bydgoszcz University of Science and Technology, 6 Bernardyńska Street, 85-029 Bydgoszcz, Poland; 2 Department of Microbiology, Ludwik Rydygier Collegium Medicum in Bydgoszcz, Nicolaus Copernicus University in Toruń, 9 Skłodowska-Curie Street, 85-094 Bydgoszcz, Poland

**Keywords:** Legionella, Legionellosis, Aquatic pathogens, Disease control, Environmental microbiology

## Abstract

An increase in the number of reports of legionellosis in the European Union and the European Economic Area have been recorded in recent years. The increase in cases is significant: from 6947 reports in 2015 to 11,298 in 2019. This is alarming as genus *Legionella*, which comprises a large group of bacteria inhabiting various aquatic systems, poses a serious threat to human health and life, since more than 20 species can cause legionellosis, with *L. pneumophila* being responsible for the majority of cases. The ability to colonize diverse ecosystems makes the eradication of these microorganisms difficult. A detailed understanding of the *Legionella* habitat may be helpful in the effective control of this pathogen. This paper provides an overview of *Legionella* environments in Europe: natural (lakes, groundwater, rivers, compost, soil) and anthropogenic (fountains, air humidifiers, water supply systems), and the role of *Legionella* spp. in nosocomial infections, which are potentially fatal for children, the elderly and immunocompromised patients.

**Graphical abstract:**

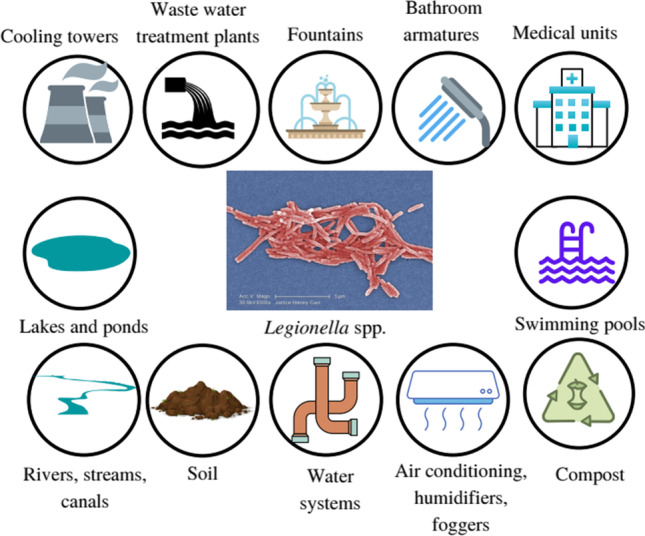

## Introduction

**Fig. 1:**
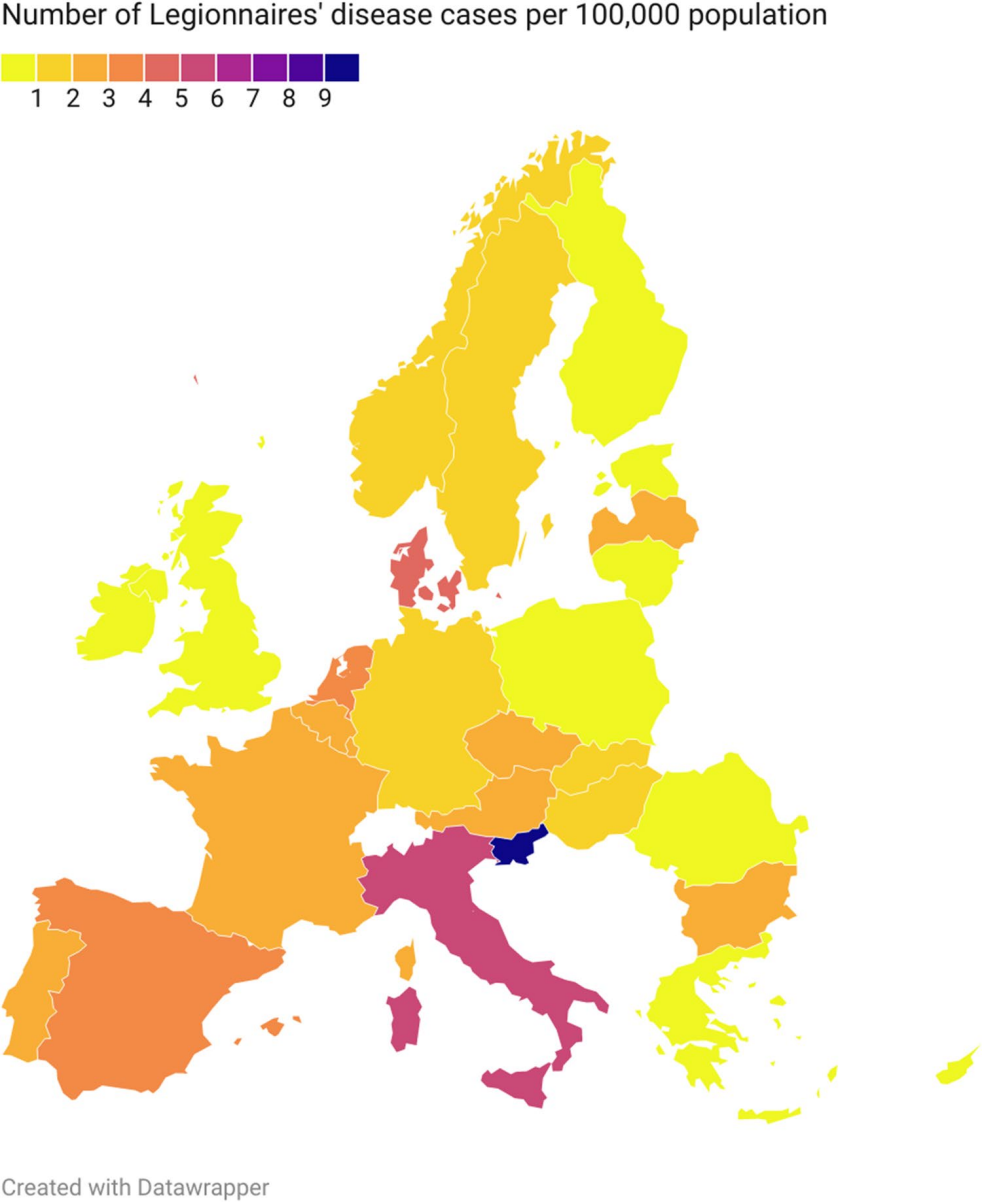
Distribution of Legionnaires’ disease cases and rates per 100 000 population by country in 2019, EU/EEA, 2021.

Legionellosis is a severe respiratory disease. An etiological factor behind it are Gram-negative rods *Legionella* spp. A milder form of infection is called Pontiac fever, with a flu-like outcome. The natural habitats of these rods are freshwater reservoirs, watercourses, moist soil, and composted material. *Legionella* bacteria also occur in man-made water systems, such as plumbing systems, air-conditioning units, bathtubs, and showers. Its presence and emitting aerosols containing pathogens may lead to an onset of illness in affected people (Kao et al. 2013; Mondino et al. 2020). So far, only one probable case of human-to-human transmission has been reported (Correia et al. 2016). The European Centre for Disease Prevention and Control (ECDC) confirmed 11,298 cases of legionellosis in 2019 on the territory of the European Union and the European Economic Area in a report published in the 2021. The ECDC report predicts an increase in the notification rate of legionellosis from 1.4 to 2.2 cases per 100,000 inhabitants in the next four years (ECDC 2019). Systematizing knowledge about the pathogen’s habitats allows for a better understanding of the microbial control and eradication mechanisms. The significant increase in the frequency of reported cases of legionellosis among EU and EEA populations, geographical differences in the frequency of cases (Fig. 1), the ubiquity of *Legionella* spp., its high resistance to environmental factors in the form of a biofilm, and some strains being pathogenic to humans call for constant environment and infrastructure monitoring for the presence of these pathogens (Schwake et al. 2021).

## Adaptive strategies of *Legionella* spp. in diverse environments

**Fig. 2:**
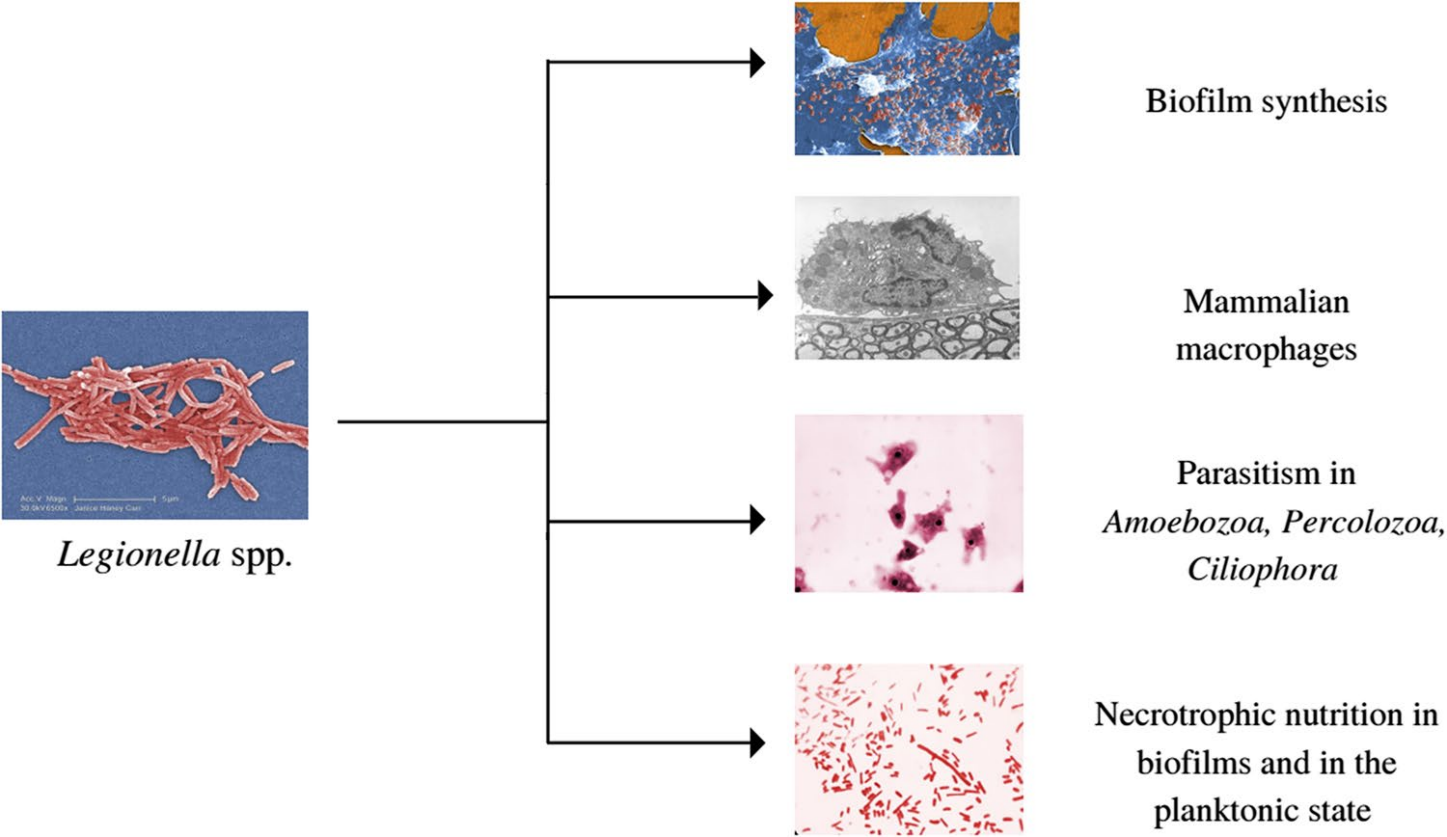
Diverse adaptation strategies of *Legionella* spp. rods (images from the Centers for Disease Control and Prevention (2022) resources, public domain).

*Legionella* spp. exhibit strong pleomorphism (taking rod-shaped, coccoid, and filamentous forms) depending on various environmental factors such as temperature, access to nutrients, presence of metabolites, and habitat (Mercante and Winchell 2015). However, despite extensive ubiquitism, these aerobic microbes are not capable of producing spore forms (Cooke and Slack 2017). Instead, these microbes have developed an ability to thrive across diverse trophic levels (Fig. 2).

Bacteria belonging to the genus *Legionella* are able to survive temperatures ranging from 0 to 68 °C, and physiological development is supported in temperatures ranging from 20 to 42 °C (Diederen 2008). *Legionella* spp. are autochthonous to natural freshwater bodies and watercourses such as lakes, rivers, moist soils, and composted plant materials (Taylor et al. 2009). Noteworthy, a study of the microbial community inhabiting glacial lakes near the Japanese research station Syowa in Antarctica, also showed the presence of *Legionella* spp. in this environment (Shimada et al. 2021). The tested bacteria were phylogenetically distinct from strains isolated from the shower nozzles at the station, demonstrating the extensive diversity and high-level adaptation. However, thermophilic populations represent a primary threat to water management, human health and life, and the environment. Tests conducted in Braunschweig, Germany, showed the presence of pathogens in tap water reaching 50°C (Lesnik et al. 2016).

*Legionella* spp. are intracellular parasites of freshwater protozoans capable of infecting and multiplying in mammalian cells by using similar mechanisms of infection (Fields et al. 2002). Colonization of both protozoan and mammalian macrophage cells occurs by phagocytosis; the pathogen enters the host cell and inhibits lysosome adhesion through the release of proteins (Taylor et al. 2009). The main organelles which *Legionella* spp. obtain their nutrients from are the endoplasmic reticulum and the mitochondrion (Tilney et al. 2001). When the level of amino acids—primary carbon source for the pathogen—drops, the bacterium produces a flagellum, which allows it to leave the cell and seek a new host (Pruckler et al. 1995). It is worth mentioning that *Legionella* species’ parasitism in protozoa allows for enhanced resistance to environmental factors (Storey et al. 2004). *Legionella* spp. also demonstrate the ability to grow necrotrophically in dead cells due to an exogenous supply of amino acids (Temmerman et al. 2006).

The ability of *Legionella* strains to exist in multispecies biofilms is a serious threat to aquatic ecosystems, as well as public health (Abdel-Nour et al. 2013). Studies on *L. pneumophila* (clinical isolate 130b) have shown the ability of the tested strain to intercalate into a biofilm formed by microorganisms such as *Klebsiella pneumoniae*, *Flavobacterium* spp., and *Pseudomonas fluorescens*. The antagonistic effect against *Legionella* was demonstrated by a monogeneous biofilm of *Pseudomonas aeruginosa* (Stewart et al. 2012). The biological dynamics of the aquatic environment reveals variable levels of relationships between permissive and antagonistic bacteria in relation to *Legionella* spp. rods. Populations of *L. pneumophila* colonizing the biofilm show high heterogeneity. Subpopulations in a “dormant” form are capable of long-term survival and pose a threat to public health (Personnic et al. 2021). Thus, the outbreaks of legionellosis are often associated with the presence of this pathogen in biofilms, even after intensive disinfection (Abu Khweek and Amer 2018; Assaidi et al. 2020). Currently, still relatively little is known about the genetic background of *Legionella* spp. biofilm formation. A recent study by Marin et al. (2022) shows that the specific gene bffA plays a signaling role in the regulatory processes involved in biofilm formation. Another example of the complexity of *Legionella* spp. environmental niches is the ability to parasitize biofilm-colonizing amoebae, resulting in reduced susceptibility of the microbes to disinfection treatments (Donlan et al. 2005). The application of materials with antimicrobial properties in water-based systems is becoming increasingly widespread. A recent study by Filice et al. (2022) shows that the implementation of a modified surface with more acidic, hydrophilic, and negatively charged properties allows for the inhibition of *L. pneumophila* adhesion to surfaces.

## Natural habitats of *Legionella* bacteria

Inland surface waters are the most significant reservoir of *Legionella* strains. Potential human exposure to *Legionellae* is most commonly associated with direct recreational activity and less frequently with bioaerosol generation by a surface water (Schwake et al. 2021). Although European inland waters reach around 20 °C during the summer season, there are many reports of the occurrence of numerous strains of *Legionella* spp. in natural aquatic environments. *Legionella* bacteria have a wide host spectrum by parasitizing a diverse group of protozoa: *Amoebozoa*, *Percolozoa*, and *Ciliophora* (Boamah et al. 2017). A study on the environmental dynamics of a pathogenic strain of *L. feeleii* in natural water bodies of central Spain showed that the presence of the bacteria without a host was found in 3.4% of raw water samples, and in 30.7% of samples as an endosymbiont of amoebae of the genus *Acanthamoeba*. The investigation shows how protozoa, which are hosts for *Legionella* bacteria, are the primary reservoir for the development and propagation of these bacteria (Vaccaro et al. 2021). *Legionella* spp. have been found in 5 natural lakes which serve as cooling systems for the Konin and Pątnów power plants in central Poland. These lakes are also used as recreation centers. *Legionella* bacteria were detected in all water samples from May to September. The highest concentration of bacteria in the biofilm was recorded in May (20.4 × 10^3^ cells per cm^-3^) and October (13.6 × 10^3^ cells per cm^-3^). It is worth noting that the average temperature of the water flowing from the power plant was 6–9 °C higher than in the lakes. According to the authors, the high concentration of microorganisms in the biofilm may be a potential threat to people resting there (Żbikowska et al. 2014). Studies conducted as part of the microbiological monitoring of the Great Mazurian Lakes (Poland) show a link between the occurrence of *Legionella* bacteria and the progressive eutrophication of lakes, the degradation of aquatic ecosystems significantly affecting human health (Grabowska-Grucza et al. 2019). The occupation of a spectrum of ecological niches by *Legionella* bacteria, such as the mining lakes of the Iberian Pyrite Belt (IPB), indicates the high environmental adaptation of this genus. The IPB lakes represent one of the largest accumulations of acid mine waste in the country. *Legionella* spp. were one of the most abundant representatives of the *Gammaproteobacteria* class there (Santofimia et al. 2013).

Rivers may also represent a reservoir of *Legionella* spp. Sampling of the French river Tech identified high abundance and diversity of *Legionella* species, even near the source of the river, where the human activity is limited. Depending on the location and month of sampling, results ranged from 100 to 583 cfu/L (Parthuisot et al. 2010). The diversity of *Legionella* spp. in the natural aquatic environment needs more understanding. This is also confirmed by Lück et al. (2021), who discovered a new strain of *Legionella* spp. in the waters of the Elbe River in Germany. Molecular studies confirmed the existence of a new strain *L. dresdenensis*.

The regional variation in the prevalence of legionellosis in the Netherlands coincides with geographical differences in drinking water origin (groundwater versus surface water). The use of surface water for drinking water production is positively correlated with high rates of Legionnaires’ disease (LD) (Den Boer et al. 2008). Also, Schwake et al. (2021) notes that conventional surface water purification methods may not be effective in controlling *Legionella* due to its long-term ability to colonize and regrow in water supply systems. However, European groundwater may also represent a reservoir of *Legionella* bacteria, despite occasional reports of their occurrence. A De Gilgio et al. (2019) study detected *Legionellae* spp. serovars in 31 (21.4%) of 145 boreholes used for irrigating plants with groundwater in southern Italy. The authors note that contaminated water applied to crop spraying can produce aerosols and pose a serious risk to agricultural workers. In fact, *Legionella* spp. show a persistent ability to colonize aquifers, as confirmed by a 7-year study in Portugal (Costa et al. 2005).

There are few data on the presence of *Legionella* bacteria outside the aquatic environment. Composting of plant-based materials is associated with a risk of *Legionella* spp. An analysis of 23 green waste composting sites in the Netherlands showed that *Legionella* was found in 97 out of 142 samples, and a strain of *L. pneumophila* was confirmed in 33 samples (Huss et al. 2020). Casati et al. (2010) confirmed the presence of *Legionellae* in 75% of tested green waste landfills in Switzerland. Contamination rates ranged from 10^3^–10^8^ cfu/g. The authors note that waste treatment facilities may pose a significant health risk by facilitating pathogen transmission. The analysis of 24 types of compost in the UK also identified the presence of *Legionella* species in 15 of them, including two strains of *L. pneumophila*. The authors call for the addition of general warnings on the packaging of commercially available compost to improve public health safety (Currie et al. 2014). A similar natural reservoir for *Legionella* is moist soil. Sprinkling *Legionella*-contaminated soil can lead to the formation of an aerosol that is a mixture of water, soil particles and pathogens. Aspiration of the bioaerosol into the lungs leads to legionellosis, following a report from the Netherlands (Boer et al. 2008). The considerable number of reports from the Netherlands may be caused by the high development of horticulture, which is an important sector of the economy. *L. pneumophila*, sequence type 47 is frequently isolated in the Netherlands; however, until now it has been obtained from clinical material; however, research has led to the isolation of the pathogen from garden soil (Schalk et al. 2014).

## Occurrence of *Legionella* bacteria in anthropogenic environments

Anthropogenic environments represent an important niche for the *Legionella* bacteria (Serrano-Suárez et al. 2013). In 2019, ECDC registered 29 outbreaks of Legionnaires’ disease from 5 countries. The number of *Legionella* spp. outbreaks in 2016–2019 ranged from 28 to 35 per year (Table 1). An outbreak is defined as an unexpected increase in the number of disease cases in a particular place and time.

**Table 1:** Number of reported Legionnaires' disease outbreaks by country in 2016-2019 (ECDC 2019).

Country	Number of outbreaks reported in:			
	2016	2017	2018	2019
France	2	3	1	3
Germany	1	1	1	4
Italy	5	7	7	9
Netherlands	8	3	6	6
United Kingdom	2	6	10	7
Spain	11	3	6	0
Hungary	3	0	1	0
Portugal	3	0	1	0
Belgium	0	2	2	0
Sweden	0	2	1	0
Finland	0	1	0	0
Luxemburg	0	0	1	0
**Total:**	35	28	37	29

Cooling towers used to reduce the temperature of water in the technological circulation may be a generator of bioaerosols containing *Legionella* bacteria. It is worth mentioning that contaminated cooling towers can spread the respirable fraction for several kilometers (Alexandropoulou et al. 2015; Walser et al. 2014). A contamination analysis of infrastructure in Greece showed that *Legionella* was found in 48.9% of the 96 tested cooling towers and 30% were classified as heavily contaminated (≥ 10^4^ cfu/L) (Mouchtouri et al. 2010). Investigations of legionellosis outbreaks in North Rhine-Westphalia in 2013 suggest that cooling towers were a possible source of contamination, due to the isolation of pathogenic *Legionella* strains from these units (Maisa et al. 2015). *Legionellae* concentration in cooling towers varies during different periods of the year, which proves the high dynamics of ecosystems and the high level of adaptability of these bacteria by adapting to harsh environmental conditions, such as periodic disinfection. A significant role in these processes is played by the ability of *Legionella* spp. to exist in biofilm (Ragull et al. 2007). Incorporating climate models and meteorological data in the study of the environmental impact of cooling towers contaminated with *Legionella* can contribute to a better understanding of pathogen transmission and detection of the outbreak’s source. An example of a successful investigation with the use of meteorological models was a study from Lidköping, Sweden. In the Swedish study, the direction of spread of the respirable fraction containing pathogens from cooling towers was determined by analyzing wind direction and strength, as well as the local topography of the area (Ulleryd et al. 2012). Fountains, especially those operating in a closed water cycle, create a favorable habitat for *Legionella*. An ecological microbiological analysis conducted as part of the investigation of the source of a legionellosis outbreak in Bresso (Italy) identified urban fountains as the most likely source of infection (Faccini et al. 2020). Another example indicating the need for preventive testing of fountains for *Legionella* spp. is the 11 cases of Legionnaires’ disease reported in northern Portugal. Conducted investigations also identified a fountain located in a city square as the most likely source of infection (Correia et al. 2001).

The hazards of *Legionella* spp. are not only restricted to outdoor infrastructure. Research in Cyprus aimed at identifying the causes of neonatal legionellosis in a hospital found that a portable air humidifier was most likely responsible for the children’s heavy exposure to the pathogens. The authors point out that the use of this kind of equipment in neonatal areas should be avoided, to reduce the potential emission of *Legionella* spp. (Yiallouros et al. 2013). In Catalonia (Spain), 12 people developed symptoms of legionellosis after visiting one of the local supermarkets. As a result of the investigation, the bacteria were detected in a fogger used in the fish section (Barrabeig et al. 2010). Bathroom fixtures can be an important reservoir of clinically important *Legionella*e strains. A fatal case in Germany is one example of an early report of the hazard of the development of *Legionella* spp. in a shower nozzle. As a result of microbiological investigations, it was concluded that a highly contaminated shower nozzle was the source of the pathogens; the number of rods was determined to be 15 × 10^3^ cfu/ml (Mühlenberg 1993). Testing of bathroom fixtures in 82 households in the UK showed that 6% of showerheads were contaminated with *Legionella* rods. Colonization of shower nozzles by pathogens was found to be associated with the shower’s duration of use and frequency of use (Collins et al. 2017). *Legionella* bacteria (including clinically relevant strains) were found in 19.44% of 36 water samples collected from beach showers in southern Italy. The authors note that especially during the summer months, considering the popularity of such places, the intense crowding, and the public accessibility of this kind of infrastructure, it may be a source of epidemics (Delia et al. 2007). Fasciana et al. (2019) point out that some water systems in prisons may also be a source of *Legionella* spp. rods. In the investigation of Legionnaires’ disease origin, 93 water samples from showers, boilers, sinks, and water tanks in 9 Sicilian prisons were tested. They found that 47.3% of the samples contained over 100 cfu/L of *L. pneumophila*, while in 4 prisons the bacterial number reached over 10^4^ cfu/L. The authors strongly recommend monitoring these types of places, due to the fact that there may be individuals from risk groups among the inmates.

Indoor water recreation areas such as swimming pools, wellness centers, whirlpools, and hot tubs require permanent sanitary monitoring because of the risk of effectively infecting significant number of people (Götz et al. 2001). Investigations into an outbreak of legionellosis in Staffordshire (UK) using molecular typing strongly suggested that the source of the contamination was a spa pool located in an enclosed commercial building. Staying in the vicinity of the contaminated pool led to the development of 21 cases of disease (Coetzee et al. 2012). A study from north-eastern France, carried out to identify the source of legionellosis in a spa, demonstrated the importance of responding to new cases quickly. While microbiological investigations were still ongoing, the entire center was closed, reducing the potential number of cases (Campese et al. 2010).

The European Agency for Safety and Health at Work recognizes wastewater treatment plants as a possible source of non-hospital *Legionella* spp. infections (Bulski 2020; Caicedo et al. 2019). The infrastructure of wastewater treatment plants creates favorable growing conditions for *Legionella* bacteria. Studies from Sweden and Finland indicate particularly strong contamination of activated sludge tanks: up to 8.0 × 10^9^ cfu/L for *L. rubrilucens* (Kusnetsov et al. 2010). Also, aeration ponds at wastewater treatment plants are a significant threat. Tests of the air near the aeration ponds at the Borregaard Ind. (Norway) showed contamination with *Legionella* spp. at up to 3300 cfu/m^3^ (Fykse et al. 2013). The use of atmospheric models provided a better understanding of pathogen propagation from wastewater treatment plants in a Dutch study (Vermeulen et al. 2021).

Public buildings, due to widespread access and high population turnover, may be an important source of *Legionella* if not effectively managed. The stagnation of water supply systems in public buildings during the SARS-CoV-2 coronavirus pandemic could impact on the growth of *Legionella* in a reduced flow environment. The authors emphasize that more research is required to investigate the colonization of stagnant water distribution systems by *Legionella* spp. (Rhoads and Hammes 2021; Almeida et al. 2021). Inadequately cleaned and managed air conditioning systems can provide an ecological niche for *Legionella* growth. Research from Ulm, Germany, highlights the difficulties associated with detecting sources of legionellosis in such systems due to the lack of regulation (Freudenmann et al. 2011). Testing of water samples from Italian retirement homes showed that the presence of *Legionella* is not rare; the pathogens were found in 36.8% of samples. This is particularly dangerous due to the fact that elderly people are in the risk group (De Filippis et al. 2018).

## *Legionella* spp. rods as a causative agent and reservoir of nosocomial infections

As it is commonly known, legionellosis affects mostly immunocompromised individuals. The higher risk groups are more likely to suffer from severe symptoms. This includes mostly immunodeficient patients belonging to elderly population with comorbidities (COPD, diabetes, renal insufficiency). LD is the most dangerous form of the legionellosis, especially amongst them. However, other groups of patients are also at risk. For example, the cases of severe pneumonia associated with *L. pneumophila* were observed also amongst neonates worldwide (Perez-Ortiz et al. 2021), and cutaneous manifestation of *Legionella* spp. in a group of immunocompromised patients (allogeneic hematopoietic cell transplantation recipients) has been revealed previously (Vaidya et al. 2020).

LD includes first of all severe pneumonia with a wide range of frequency and severity of disseminated pulmonary symptoms. In general, the manifestation includes fever, headache, myalgia, and pneumonia-related signs (cough—initially non-productive, then usually productive). However, the symptoms might be also extrapulmonary, including diarrhea, nausea, or confusion. The incubation period of LD usually lasts from 2 days to 2 weeks (Cunha et al. 2016; Burillo et al. 2017; Tomaskovic et al. 2022) and the estimated cost of LD treatment in hospitalized patients in the USA reaches over USD 433 million (2012) (Falkinham 2020).

Of note, generally healthy people may also develop the disease, usually with milder symptoms. Regardless of the patient’s state, without appropriate diagnostics, the infection may lead to the development of multi-organ failure and even death in both groups. Unfortunately, LD is likely to be under-diagnosed, due to the lack of reliable and disseminated diagnostic approaches but also dissemination and variety of pathogenic *Legionella* spp. strains (Feng et al. 2021). Worth mentioning, the bacteria are relatively hard to culture and require fastidious growth conditions, specific tests for identification, and antimicrobial susceptibility testing procedures. The obstacles results also from the overall surveillance system insufficiencies as well as the variability of disease definitions and the applied diagnostic approaches used worldwide (Cunha et al. 2016; Burillo et al. 2017; Tomaskovic et al. 2022).

The patients with laboratory-confirmed disease (positive classic sputum culture, non-culture urine antigen test, or PCR) (Metlay et al. 2019; Kawasaki et al. 2022; Miyashita 2022) are commonly treated with the antibiotics active against *Legionella* spp. (usually fluoroquinolone – levofloxacin or macrolide – azithromycin). These antimicrobials are currently preferred due to the bactericidal features: satisfactory lung tissue penetration, high intracellular concentrations of the drug, and potency to target multiple *L. pneumophila* serogroups responsible for the majority of the cases (Metlay et al. 2019; Miyashita 2022).

In the case of extrapulmonary *Legionella* spp. infections, the patients are commonly treated with fluoroquinolones (e.g., levofloxacin), accompanied with routine surgical procedures (incision or drainage) (Metlay et al. 2019; Miyashita 2022.

Thus, *Legionella* spp. rods might be the causative agents of both hospital-acquired pneumonia (HAP), also called nosocomial infections, and the community-acquired pneumonia (CAP) cases, that origin outside the hospital. Since natural soil and waters are the ultimate sources of *Legionella* spp. strains, they are quite easily adapted to grow and survive in flowing water systems. Moreover, they are particularly adapted to survival, persistence, and growth in the human-built environment, particularly in hospital structure. *L. pneumophila*, as the colonist of human-engineered water systems tolerates selection pressure for growth under low nutrient conditions. Thus, these opportunistic pathogens are quite able to survive and proliferate also in plumbing in hospitals. As an example, 4 years of Italian monitoring of *Legionella* spp. in hospital water systems demonstrated that 49% of 253 water samples were positive for *Legionella* bacteria (Arrigo et al. 2022). Water in premise plumbing is regularly heated by water heater (e.g., to 50 °C) and distributed throughout the whole structure (hospital or healthcare facility). But eventually it cools down to an optimal temperature for bacteria growth (to 25–37 °C). Thus, recirculating hot water systems in hospitals provide finally optimal temperature for *L. pneumophila*, and other waterborne pathogens, growth. It refers especially to hospitals where some wards or their wings are not always occupied, water residue within that portion of the distribution system. Oxygen therapy devices (nebulizers, oxygen masks) may also be a secondary carrier of infection (Allegra et al. 2016).

Pathways of waterborne pathogens transmission in general include aerosolization, drinking water, and direct contact with water (Falkinham 2020). Exposure to any of the mentioned three pathogen transmission routes can occur in both natural and anthropogenic environments. In this connection, however, nosocomial infections are of particular importance. Immunocompromised and other hospital patients are particularly exposed to them through all three ways during stays in hospitals and other healthcare centers. Aerosolization refers to the generation of water droplets from water, most commonly inhaled, leading to lung infections as the water could carry *L. pneumophila*. In addition, due to room showers and sinks, patients are exposed to aerosols generated by therapy baths, patient manipulations, and medical equipment. Medical equipment that has been shown to be aerosol sources of waterborne bacteria also include bronchoscopes and heater–coolers (e.g., water heating and cooling devices used in operating rooms to cool patients and warm blood). Unsurprisingly, hospitals and other healthcare facilities can be designed to avoid creating ideal growth conditions for waterborne pathogens, but it is usually quite expensive (Falkinham 2020).

Although the antimicrobial treatment schemes in the *Legionella* spp. infection are well known, the mortality rate in the case of LD remains quite high (Phin et al. 2014; Tomaskovic et al. 2022). For HAP it is generally higher in comparison to the CAP, ranging between 15 and 34% (Phin et al. 2014; Tomaskovic et al. 2022). Noteworthy, healthcare-associated infections (HAIs) are currently one of the most common patient complications, affecting in the developed countries each year approximately 7% of patients. But it is the rise of antimicrobial resistant (AMR) bacteria that has been identified as one of the biggest global health challenges, resulting in an estimated 23.000 deaths in the USA annually. Environmental reservoirs for AMR typical aerobic bacteria (e.g., bed rails, light switches, doorknobs) have been previously identified and addressed with infection prevention guidelines. However, water and water-related devices are often overlooked as potential sources of HAI outbreaks and AMR source. The role of water and common water-related devices also in the transmission of AMR bacteria responsible for HAIs is discussed recently since AMR strains of waterborne pathogens, including *Legionella* spp., are commonly isolated. Continuous monitoring of water systems and observation of changes in the resistance pattern is strongly recommended (Rahimi and Vesal 2017; Hayward et al. 2020; Pappa et al. 2020).

Great efforts worldwide are put into the establishing of unified diagnostic procedures as well as development of a specific and truly active vaccine against *Legionella* spp. infections; however, a sufficiently effective and non-toxic vaccine is still missing. Therefore, more reliable, and detailed microbiological investigation approaches, based on the available biochemical and/or molecular methods are required to improve the diagnostic accuracy and treatment schemes to reach a deeper insight into the pathomechanism of the *Legionella* spp. infections and their eradication (Phin et al. 2014; Legionnaires Disease Guidelines 2022; Chauhan and Schames; 2021).

## *Legionella* spp. control strategy

Analyzing the diverse habitats of *Legionella* spp. allows for the implementation of methods that reduce the possibility of spreading these pathogens. In the study by Spies (2021) on microbiological monitoring of cooling towers, a statistical predictive tool was designed. The application of correlation analyses of retrospective microbiological data and parameters, water temperature or redox potential, allowed for an effective prediction of the potential occurrence of hazards. As Sciuto et al. (2021) note, routine water testing is essential; however, creating a water system management plan based on this factor alone may not be sufficient. Modifying the water system by implementing modern coatings to inhibit biofilm formation by *Legionella* spp. can reduce the spread of these pathogens effectively (Filice et al. 2022). Also, monitoring and reducing the presence of protozoan hosts for *Legionella* rods may prove helpful in reducing the risk to humans (Nisar et al. 2020). Water testing for optimum iron concentration for *Legionella* spp. proved to be an effective indicator in a study by Vittal et al. (2021), where an iron concentration of 300 mg Fe/L demonstrated a positive correlation with the presence of *Legionella* spp. bacteria. Another critical element is to counteract the long-term stagnation of water systems, as was the case during the SARS-CoV-2 coronavirus pandemic. The reopening of water systems after stagnation should be associated with a detailed microbiological analysis to ensure health safety (Palazzolo et al. 2020). In case of the natural environment, counteracting the eutrophication processes of water reservoirs may contribute to reduction of *Legionella* spp. abundance in the natural aquatic environment and, consequently, improving public safety (Grabowska-Grucza et al. 2019).

The implementation of these methods in combination with classical physical and chemical disinfection procedures and the maintenance of a sanitary regime will positively influence public health safety.

## Summary

This paper shows examples of the diverse environments that may be colonized by *Legionella* spp. and the ability of the pathogens to respond rapidly to environmental alterations. Reports of the presence of the microorganisms came from Spain, Portugal, France, Germany, Switzerland, the Netherlands, Poland, Sweden, Norway, Finland, the UK, Italy, Greece, and Cyprus. The isolation of clinically important strains from both natural and anthropogenic environments demonstrates that these bacteria, due to their ubiquitism, pose a serious threat to public health. The paper also describes the role of *Legionella* spp. in hospital infections. Some of the presented environmental studies were based on molecular detection methods, which shows that it is an effective tool in microbiological diagnosis, especially against strains difficult to grow in vitro. Understanding the modes of transmission and environmental niches of *Legionellae* spp. allows effective identification of the pathogen source and therefore appropriate prophylaxis. This is particularly important in view of the increasing frequency of reported cases of legionellosis in Europe.

In conclusion, monitoring the presence of *Legionella* spp. as well as the factors promoting its occurrence, together with the simultaneous implementation of prophylactic interventions and predictive tools based on statistical analysis, may be the key to successfully control the presence of *Legionella* spp. representatives in different types of environments.

## Data Availability

Not applicable.
